# Distribution and chemotactic mechanism of CD4^+^ T cells in traumatic tracheal stenosis

**DOI:** 10.1002/iid3.916

**Published:** 2023-08-09

**Authors:** Tingmei Feng, Yan Chen, Jinmei Wei, Sen Tan, Liu Guangnan

**Affiliations:** ^1^ Guangxi Medical University Nanning China; ^2^ Department of Respiratory Medicine The Second Affiliated Hospital of Guangxi Medical University Nanning China

**Keywords:** CD4^+^ T, chemokines, traumatic tracheal stenosis

## Abstract

A systemic and local inflammatory immune imbalance is thought to be the cause of traumatic tracheal stenosis (TS). However, with CD4^+^ T lymphocytes being the predominant immune cells in TS, the mechanism of action and recruitment has not been described. In our research, using flow cytometry, ELISA, immunofluorescence, and Transwell chamber assays, the expression, distribution, and potential chemotactic function of CD4^+^ T cells in TS patients were examined before and after treatment. The results showed that the untreated group had significantly more CD4^+^ T cells and their secreted TGF‐β1 than the treated group. Additionally, the untreated group's CD4^+^ T cells showed a significant rise in CCL22 and CCL1, as well as a larger proportion of CCR4 and CCR8. CD4^+^ T cells and CD68^+^ macrophages located in TS also expressed CCL1 and CCL22. In vitro, anti‐CCL1 and anti‐CCL22 can partially block the chemoattractant effect of TS bronchoalveolar lavage (BAL) on purified CD4^+^ T cells. The findings of this study indicated that TS contained unbalanced CD4 immune cells that were actively recruited locally by CCR4/CCL22 and CCR8/CCL1. As a result, it is anticipated that CD4 immune rebalancing can serve as a novel treatment for TS.

## Graphical abstract

Our study indicated that traumatic tracheal stenosis contained unbalanced CD4 immune cells that were actively recruited locally by CCR4/CCL22 and CCR8/CCL1. CD4 immune cells and their subtypes Tregs may participate in the process of tracheal fibrosis through secretion of TGF‐β1.

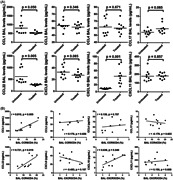

## 1 INTRODUCTION

Traumatic tracheal stenosis (TS) refers to the proliferation of granulation and inflammation and scar tissue in the trachea caused by tracheal intubation, tracheotomy, trauma, and other factors. Patients typically experience a narrowing of the tracheal lumen along with clinical dyspnea, coughing, and wheezing. This lowers their quality of life and, in extreme cases, can result in death from respiratory failure brought on by airway obstruction.^1^, ^2^, ^3^, ^4^ TS can be treated by raditional treatment methods: surgery and bronchoscopic procedures,^5^, ^6^ but numerous patients still develop granulation tissue and restenosis after interventions, and the recurrence rate of postoperative TS reaches as high as 40%–70%.^7^ Recent studies have shown that tracheal tissue engineering and transplantation may provide important help for patients with severe cicatricial stenosis.^8^, ^9^

The cause of TS is complex. Recent findings have shown that immune cell dysregulation is a significant contributor to TS.^10^, ^11^, ^12^ Previous research has reported that B and T cells are involved in mediating granulation tissue formation in laryngotracheal stenosis, while CD4^+^ T cells act a pivotal part in the fibrosis process.^12^ There are various subtypes of CD4^+^ T lymphocytes. Prior studies have revealed that the Th1/Th2/Th17/Treg lineages can be linked to one another, change into different lineages, and play distinct roles in the progression of fibrosis.^13^ Th1 cells are primarily thought to have an antifibrotic effect in related studies^14^; nevertheless, they can also be detrimental to bone regeneration^15^ and fibrotic disorders.^16^, ^17^ There is controversy around the function of regulatory T cells (Tregs) in fibrosis and their connection to a specific disease model.^18^ But none of them have been reported in TS.

Transforming growth factor (TGF)‐β is a key factor in fibrosis. Studies have revealed that a huge portion of TGF‐β accumulates locally in TS, causing excessive proliferation of fibroblasts, increased collagen synthesis, extracellular matrix deposition, and fibrosis through the action of TGF‐β/Smad2/3, ERK1/2, and other pathways, and eventually leading to airway stenosis.^19^, ^20^, ^21^ Recent findings have also strongly suggested that TGF‐β is critical for developing and differentiating Forkhead box protein 3 (FoxP3) Tregs.^22^ Tregs are mostly produced in the thymus (tTreg), but they can also be produced extrathymically at peripheral sites (pTreg) or induced in cell culture (iTreg) in the presence of transforming growth factor (TGF‐β).^23^ All Treg cell types express FoxP3, especially the thymic population (tTreg), and FoxP3 serves as a spectrum‐specific and key transcription factor for maintaining Treg cell phenotype and suppressor function.^24^, ^25^, ^26^, ^27^ This study hypothesizes the presence of local immune cell infiltration and imbalance in TS and focuses on the secretion, aggregation and relationship with TGF‐β1 of CD4^+^ T cells and its subtype Tregs before and after TS treatment.

According to studies, CD4^+^ T cells create the environment necessary for monocytes to differentiate into fibroblasts and participate in the negative control of fibrosis by secreting cytokines.^28^ Additionally, a variety of inflammatory cytokines and chemokines are produced locally by macrophages, which contribute to the development of the inflammatory response, fibrosis, and abnormal wound healing. When there is an imbalance of Th1/Th2 cells in tissue fibrosis, Th1 cells secrete cytokines such as INF‐γ, which is mainly involved in the inhibition of fibrosis,^29^ while Th2 cells mainly secrete IL‐4, which promotes the development of fibrosis.^30^ CXCR3 and CCR5 are predominantly expressed on the surface of polarized Th1 cells, whereas CCR4 and CCR8 are mainly distributed in Th2, mast cells and eosinophils. Therefore, different chemokine types can be used to evaluate the Th1/Th2 balance.^31^ These chemokine receptors expressed on lymphocytes can be gained and detected in cells obtained by bronchoalveolar lavage (BAL) and tracheal granulation tissue biopsy. According to a larger body of data on pulmonary fibrosis, CXCR3 and its ligands CXCL11 (IFN‐inducible T‐cell α‐chemoattractant, I‐TAC)^32^ and CXCL10 (IFN‐γ‐inducible protein, IP‐10)^33^ play an important role in bleomycin‐induced lung fibrosis models in mice.^34^ Belperio et al.^35^ found that the bleomycin‐induced lung fibrosis model in mice was associated with the CCR4 receptor and its agonizts CCL17 (thymic and activation‐regulated chemokine, TARC) and CCL22 (macrophage‐derived chemokine, MDC). In a recent report, it was discovered that the CCR5 ligands CCL3 (macrophage inflammatory protein (MIP)‐1) and CCL4 (MIP‐1) are both considerably raised in BAL fluid of patients with idiopathic pulmonary fibrosis and have an essential impact in animal models of lung fibrosis.^36^, ^37^ Furthermore, Liu et al.^38^ confirmed that CCL1 was recruited into the lung through fibroblasts expressing its receptor CCR8 and triggered the AMFR‐SPPY1 pathway, thereby promoting the development of pulmonary fibrosis. However, little is known about cellular immune function in TS, and there are no data on the specific role played by relevant chemokines in TS. This study aims to assess the ratios of CD4^+^ T cells with the receptors CXCR3, CCR4, CCR5, and CCR8 in BAL fluid and synchronized peripheral blood of TS patients before and after treatment.

## 2 MATERIALS AND METHODS

### 2.1 Subjects

The study protocol was approved by the Ethics Committee of the Second Affiliated Hospital of Guangxi Medical University (Nanning, China), and written consent was obtained from the subjects. Ten patients with TS were recruited, and they all had tracheal injuries due to tracheal intubation or tracheotomy. The proliferative scar tissue was pathologically confirmed. The control group was 10 patients with peripheral pulmonary nodule operation. BAL fluid and blood specimens were collected before and after comprehensive treatment of TS patients (interventional pulmonology procedures, systemic anti‐infection, sputum removal, and rehabilitation exercises), and tracheal tissues of untreated TS patients and control group were collected simultaneously.

### 2.2 General detection

Pulmonary function tests, including vital capacity (VC), forced vital capacity (FVC), forced expiratory volume in the first second (FEV1), the forced expiratory volume in the first second in percent predicted values (FEV1/Pred%), and percent of forced vital capacity exhaled in the first second (FEV1/FVC%), were performed for the 10 controls and the 10 patients before and 1 month after the comprehensive treatment. In addition, blood routine, blood gas analysis, and inflammatory indexes, including arterial partial pressure of oxygen (PO2), partial pressure of carbon dioxide oxygen (PCO2), oxygenation index (PaO2/FiO2), C‐reactive protein (CRP), erythrocyte sedimentation rate (ESR), total leukocyte count (WBC), neutrophil count (NEU), and lymphocyte count (LYM) were assessed for the control group and all the patients before and after treatment.

### 2.3 Sample collection and processing

Tracheobronchial stenosis granulation tissue was collected during bronchoscopy and normal bronchial mucosa tissue was collected during surgery. BAL samples were collected by placing the bronchoscope over the tracheal stenosis of the patient before and after TS treatment, and quickly injecting the total amount of 37°C sterilized normal saline about 150–200 mL, and recovering 60–120 mL with a recovery rate of 40%–60%, and they were processed in accordance with the European Respiratory Society (ERS) guidelines.^39^ Simultaneously, 20 mL blood was drawn. BAL samples were immediately placed in ice and then centrifuged at 1200*g* for 5 min, and the BAL supernatant was collected for subsequent ELISA and chemotactic assays. The cell pellet of BAL fluid and blood was resuspended in phosphate buffered saline (PBS), and mononuclear cells were isolated by Ficoll‐Hypaque gradient centrifugation (Solarbio) within 1 h.

### 2.4 Flow cytometry

Dead cells were stained with BD Horizon™ Fixable Viability Stain 700. Moreover, the expression of markers on T cells from BAL fluid and blood was stained with antibodies, including anti‐human CD3‐APC/Cy7, CD25‐APC, CD4‐BB515, CXCR3‐APC, CCR4‐PE, CCR5‐APC, and CCR8‐PE (BD Biosciences). Cells were acquired with a flow cytometer (Beckman Coulter), and data were analyzed with FlowJo 10.

### 2.5 Enzyme‐linked immunosorbent assay (ELISA)

The levels of CCL1, CCL3, CCL4, CXCL9, CXCL10, CXCL11, CCL17, and CCL22 were evaluated in undiluted BAL samples between the untreated and treated patients with commercial immunoassays (Fankew) according to the manufacturer's instructions.

### 2.6 Western blot detection

Western blotting was performed using standard procedures. Total protein was isolated from tracheal tissues using radioimmunoprecipitation assay buffer (high)‐ phenylmethylsulfonyl fluoride (RIPA‐PMSF; SolarBio). Subsequently, total protein content was determined using a BCA assay kit (SolarBio). Equal amounts of protein were electrophoresed by SDS‐PAGE and then transferred to nitrocellulose membranes (Merck Millipore). Five percent skim milk was inhibited for 1 h and incubated for 12 h at 4°C with the following primary antibodies: COL1A1 (ab260043), CD4 (ab133616), CD25 (ab231441), and TGF‐β1 (ab215715), all antibodies were purchased from Abcam at a dilution of 1:1000. After three washes in TBST, they were incubated with the corresponding secondary antibodies for 1 h at room temperature. Finally, immunoblots were detected using an electrochemiluminescence plus reagent kit (Biosharp), and densitometric analysis of each target protein band was performed using ImageJ software (NIH).

### 2.7 Immunohistochemistry

Formalin‐fixed and paraffin‐embedded specimens were collected from three patients in the control group and TS group. Hematoxylin and eosin (H&E) staining, Masson's trichrome staining, and immunohistochemical detection of CD4, CD25, and TGF‐β1 were performed, respectively. The primary antibody was purchased from Abcam (USA) with the same clone number as Western blotting. Immunostained sections were imaged at high power (400x) and three areas of intense inflammation found at low power (100x) were imaged. Percentage of positive‐stained cells was determined by diving the number of positive‐staining cells by total cells in each high‐power field (hpf).^11^

### 2.8 Immunofluorescence assay

The cell suspensions were obtained by BAL centrifugation, dehydrated, embedded in paraffin, sectioned in paraffin, dewaxed, rehydrated using an alcohol gradient, antigen repaired, and closed. They were then incubated with CCL1 (Biorbyt), CCL22 (Biorbyt), CD4 (ProteinTech Group), and CD68 (ProteinTech Group) antibodies at 4°C overnight, followed by incubation with the corresponding fluorescent substance‐labeled secondary antibodies for 1 h. After mounting with DAPI (Solarbio), immunofluorescence images were recorded using an orthofluorescence microscope with phase contrast (Nikon).

### 2.9 Quantitative real‐time polymerase chain reaction (RT‐PCR)

RNA was extracted and purified from the 10 TS patients and the 10 control subjects using an RNA isolation kit (Vazyme Biotech). As a key Treg cell transcription factor,^23^, ^40^ the mRNA level of FoxP3 was analyzed using quantitative RT‐PCR. The cycle threshold (CT) value of FoxP3 was normalized against GAPDH (Δ*C*_T_) for all samples. Gene expression was displayed as the relative fold change (2−ΔΔ*C*_T_). All samples were investigated in triplicate.

The primer sequences used in the quantitative polymerase chain reaction (qPCR) were as follows: Gene: FoxP3 (Forward: 5ʹ‐ AGTTCCTCCACAACATGGACTACT‐3ʹ; Reverse: 5ʹ‐ ATTGAGTGTCCGCTGCTTCTCT ‐3ʹ). Gene GAPDH: (Forward: 5ʹ‐ ACATCGCTCAGACACCATG‐3ʹ; Reverse: 5ʹ‐ TGTAGTTGAGGTCAATGAAGGG‐3ʹ). All the reagents were purchased from Takara.

### 2.10 Cell isolation and transwell chamber assay

CD4^+^ T cells were isolated by magnetic‐activated cell sorting (MACS) based on negative selection using the CD4^+^ T‐cell isolation kit (Miltenyi Biotec). The purity of CD4^+^ T cells was typically >96%, as detected by flow cytometry.

For chemotaxis assays, a 6.5 mm Transwell with a 5.0 µm pore polycarbonate membrane insert (Corning Costar) was used. BAL supernatants were collected as described previously. The purified CD4^+^ T cells were cultured in a Transwell chamber, and BAL supernatants and neutralizing antibodies against the chemokines were cultured in a lower layer culture plate. Grouping was as follows: PBS control group, BAL supernatant group, BAL + anti‐CCL1 (Thermo Fisher Scientific) group, BAL + anti‐CXCL10 (Thermo Fisher Scientific) group, and BAL + anti‐CCL22 (Thermo Fisher Scientific) group. These groups were incubated in an incubator at a constant temperature for 16 h without or with neutralizing antibodies against the chemokines. The cells on the upper surface of the membrane were removed with a cotton swab, and the cells that migrated to the lower side were fixed with methanol and stained with 0.1% crystal violet. The membrane was visualized with an inverted microscope at ×200 magnification. Cell counting was performed using the ImageJ software. Four representative visual fields were selected for each membrane, and six separate experiments were performed.

### 2.11 Statistical tests

SPSS V.26.0 (SPSS Inc) was employed for the statistical tests. Data are expressed as the mean ± SEM. Within‐group comparisons before and after treatment were made using paired *t* tests or Wilcoxon's signed‐rank tests as appropriate. Between‐group data were analyzed using independent *t* tests or Mann–Whitney *U* tests as appropriate. Statistical differences between groups were evaluated using one‐way ANOVA followed by the Newman–Keuls test for normally distributed continuous variables. Correlations were assessed with Spearman's rank tests. *p* < .05 was considered statistically significant.

## 3 RESULTS

### 3.1 General information

**Figure 1:**
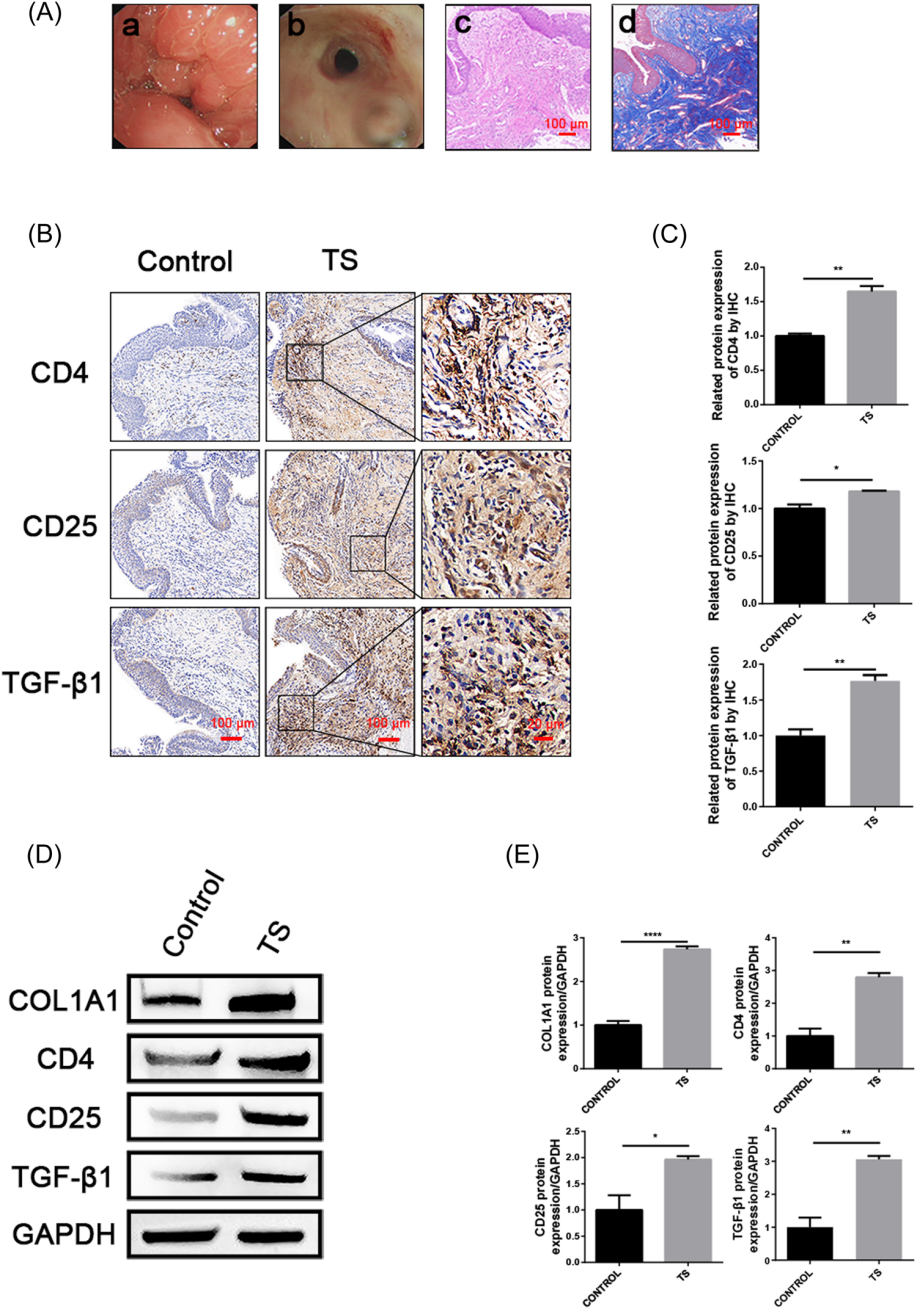
Significantly greater expression of CD4, CD25, and TGF‐β1 were observed in TS patients with untreated groups compared to controls. The image in a and b of (A) showed the hyperplasia of tracheal granulation tissue after tracheotomy and the narrowing of the scar after tracheal intubation under tracheoscopy, respectively. The image in c and d of (A) showed that the untreated TS group underwent HE staining and Masson staining, respectively, and a large amount of collagen hyperplasia was observed in tracheal mucosa under the microscope. Scale bar = 100 μm. (B and C) Immunohistochemical (IHC) assessment of CD4, CD25, and TGF‐β1(brown) in control and TS tracheal granulation tissue specimens. In untreated TS tracheal specimens, CD4, CD25, and TGF‐β1 expressions were elevated. Scale bar = 100 μm. The levels of COL1A1, CD4, CD25, and TGF‐β1 proteins were assessed by western blotting and quantified by densitometric analysis (D) and (E). Between‐group data were analyzed using independent t tests or Mann–Whitney *U* tests, *n* = 3 per group. *p < .05, **p < .01, ***p < .001. TS, tracheal stenosis.

The 10 patients with TS were caused by tracheal intubation (*n* = 6) and tracheotomy (*n* = 4), as shown in Figure 1A, and the 10 control subjects had normal functional data. All the patients with TS underwent the comprehensive treatment, during which pulmonary function (FEV1 and FEV1/Pred%), blood gas analysis (PO2 and PaO2/FiO2), and lymphocyte count (LYM) improved, and ESR decreased. Compared to the control subjects, the untreated TS patients showed a decrease in lung function indexes, blood gas analysis indexes, and lymphocyte count, and an increase in CRP and ESR, all of which were statistically significant, as shown in Table 1.

**Table 1:** Characteristics of the patients enrolled.

	Pre‐T (*n* = 10)	Post‐T (*n* = 10)	CTRL (*n* = 10)
Age, year	37.5 ± 4.5	37.5 ± 4.5	30.3 ± 1.8
Sex, M/F	7/3	7/3	6/4
VC, mL	2.6 ± 0.2**	2.7 ± 0.2	3.6 ± 0.1
FVC, mL	2.6 ± 0.2**	2.7 ± 0.2	3.6 ± 0.1
FEV1, mL	1.8 ± 0.1* ^,^ **	2.0 ± 0.1	3.2 ± 0.1
FEV1/Pred, %	55.5 ± 5.9* ^,^ **	59.5 ± 5.9	91.3 ± 0.6
FEV1/FVC, %	70.3 ± 3.7**	76.3 ± 4.1	89.4 ± 1.1
PO2, mmHg	78.6 ± 3.0* ^,^ **	91.2 ± 1.9	97.2 ± 0.5
PCO2, mmHg	39.1 ± 1.2	39.7 ± 1.2	37.5 ± 0.6
PaO2/FiO2, mmHg	354.8 ± 21.6* ^,^ **	434.2 ± 9.0	462.9 ± 2.3
CRP, mg/L	18.9 ± 5.0**	7.5 ± 1.8	2.8 ± 0.3
ESR, mm/h	65.3 ± 8.6* ^,^ **	15.8 ± 1.8	7.2 ± 1.3
WBC, 10^9^/L	9.1 ± 1.3	7.5 ± 0.2	7.2 ± 0.2
NEU, 10^9^/L	7.0 ± 1.3	4.6 ± 0.2	4.1 ± 0.2
LYM, 10^9^/L	1.2 ± 0.1* ^,^ **	2.1 ± 0.2	2.3 ± 0.2

*Note*: Data are shown as mean ± SEM.

Abbreviations: CTRL, healthy control subjects; Pre‐T, pretreatment; Post‐T, posttreatment.

* *p* < .05 vs. Post‐T

** *p* < .05 vs. CTRL.

### 3.2 CD4, CD25, and TGF‐β1 expression is increased in the untreated patients with TS

In the untreated group, hematoxylin and eosin (H&E) staining and Masson's trichrome staining showed a large number of nodular collagen fibrous tissue hyperplasia in the tracheal mucosa. The diagnosis was hypertrophic scar tissue (Figure 1A). It was found in the experiment that compared with the normal control group, the collagen fibers in the tracheal tissue of TS patients in the untreated group were increased, and the protein expression of COL1A1 was significantly increased, which both indicated that there was obvious fibrosis in the tracheal tissue of TS patients in the untreated group. Immunohistochemistry and western blot analysis demonstrated a statistically significant increase in CD4, CD25, and TGF‐β1 in the tracheal tissues of TS patients in the untreated group compared to the healthy control group (Figure 1B–E, *p* < .05).

### 3.3 Comparison of CD4^+^ T cells and Tregs expression in the untreated groups, treated groups, and control groups

**Figure 2:**
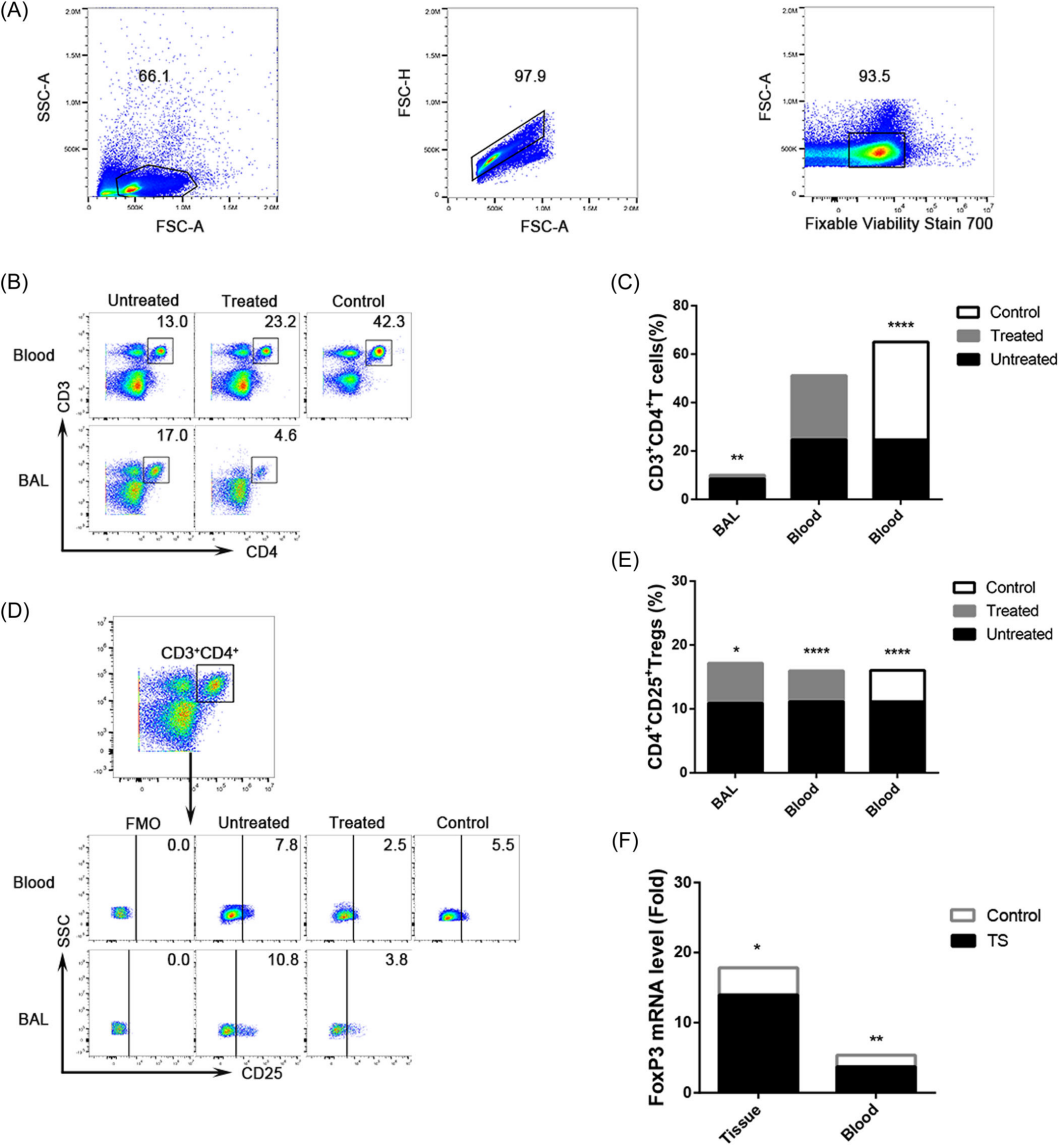
The expression of CD3^+^CD4^+^ T cells and Tregs in BAL fluid and peripheral blood of the untreated groups, treated groups, and control subjects. (A, B, D) Representative flow cytometric dot plots of CD3^+^CD4^+^ T cells and Tregs in BAL fluid, blood, and control. (C and E) Comparisons of percentages of CD3^+^CD4^+^ T cells and Tregs in BAL fluid, blood, and control (*n* = 10). (F) qPCR analysis of FoxP3 in the tissue and peripheral blood of the patients and control subjects. Within‐group comparisons were made using paired *t* tests or Wilcoxon's signed‐rank tests. Between‐group data were analyzed using independent *t* tests or Mann–Whitney *U* tests. The bar graph shows the mean. *p < .05, **p < .01, ***p < .001, and ****p < .000. BAL, bronchoalveolar lavage.

This research chose CD3^+^ and CD4^+^ cells to define CD4^+^ T cells (Figure 2B) and CD3^+^, CD4^+^, and CD25^+^ to define Tregs (Figure 2D) during flow cytometry. It was found that the untreated patients had a significantly higher expression of CD4^+^ T cells and Tregs in BAL fluid and Tregs in peripheral blood than the treated patients and the control subjects (for CD4^+^ T cells and Tregs in BAL fluid, *p* = .007 and *p* = .015, vs. the treated patients; for CD4^+^ T cells and Tregs in peripheral blood, *p* = .522 and *p* = .000, vs. the treated patients, *p* = .000 and *p* = .000 vs. the control subjects; Figure 2C,E).

It was further discovered that the expression of FoxP3 mRNA, a crucial transcription factor of Tregs, was higher in tissue and peripheral blood of the untreated patients than that of the control subjects (*p* = .018 and *p* = .006, respectively; Figure 2F).

**Figure 3:**
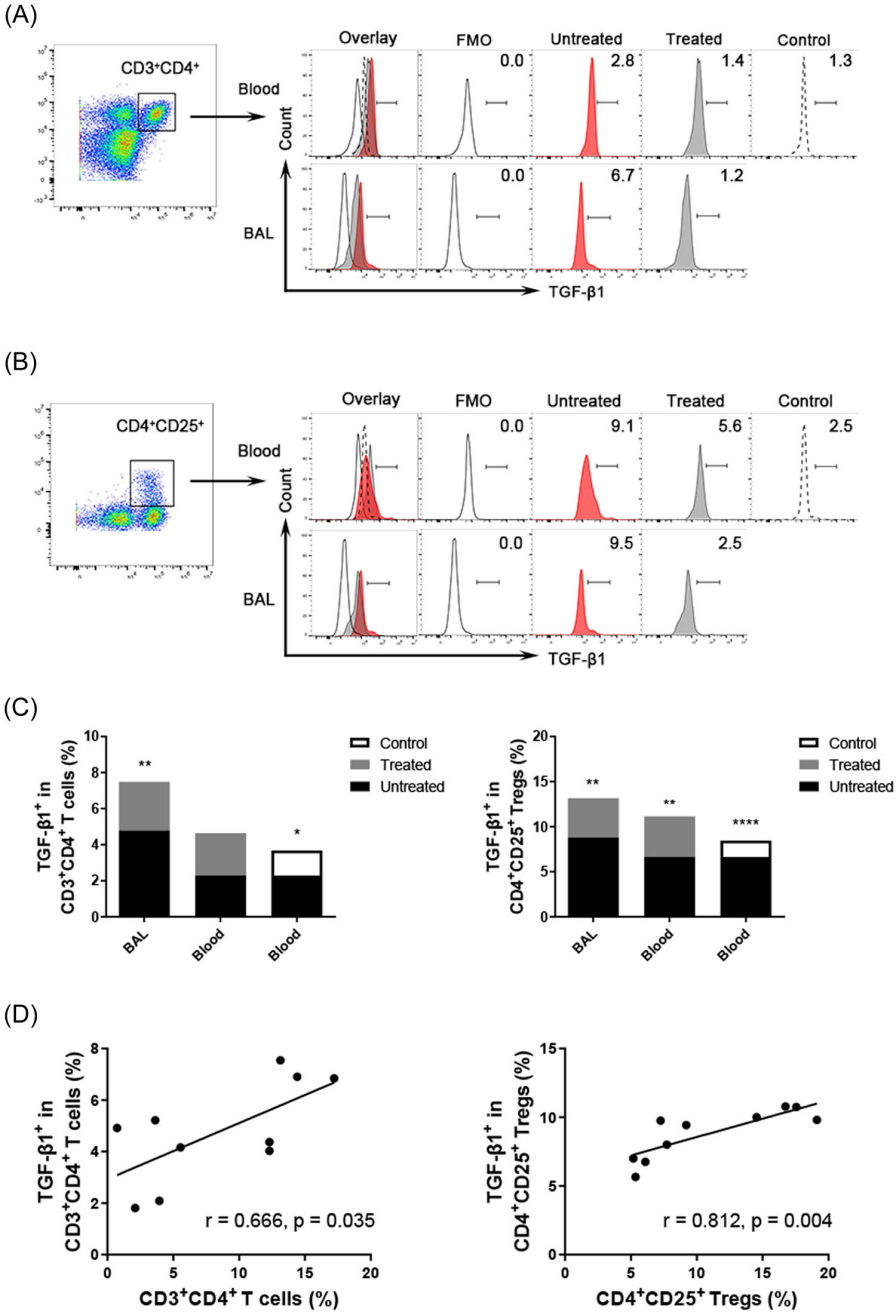
The expression of TGF‐β1^+^CD4^+^ T cells and TGF‐β1^+^ Tregs in BAL fluid and peripheral blood of the untreated groups, treated groups, and control subjects. (A and B) Representative flow cytometric dot plots of TGF‐β1^+^CD4^+^ T cells and TGF‐β1^+^ Tregs in BAL fluid, blood, and control. (C) Comparisons of the percentages of TGF‐β1^+^CD4^+^ T cells and TGF‐β1^+^ Tregs in BAL fluid, blood, and control (*n* = 10). (D) Correlation between TGF‐β1^+^CD4^+^ T cells and CD3^+^CD4^+^ T cells and correlation between TGF‐β1^+^ Tregs and Tregs in BAL fluid of the untreated groups. Within‐group comparisons were made using paired *t* tests or Wilcoxon's signed‐rank tests. Between‐group data were analyzed using independent *t* tests or Mann–Whitney *U* tests. Correlations were determined by Spearman's rank correlation coefficients. The bar graph shows the mean. *p < .05, **p < .01, ***p < .001, and ****p < .000. BAL, bronchoalveolar lavage.

As shown in Figure 3, the cytokine TGF‐β1 was highly expressed in CD3^+^CD4^+^ T cells and Tregs in BAL fluid and Tregs in peripheral blood of the untreated patients compared with the treated patients and the control subjects (for CD4^+^ T cells and Tregs in BAL fluid, *p* = .009 and *p* = .002, vs. the treated patients; for CD4^+^ T cells and Tregs in peripheral blood, *p* = .913 and *p* = .004, vs. the treated patients, *p* = .012 and *p* = .000 vs. the control subjects; Figure 3A–C).

Taking into account all the patients without the comprehensive treatment, the levels of cytokine TGF‐β1 had a strong positive correlation with the expression of CD4^+^CD25^+^ BAL cells (*r* = .812, *p* = .004) and a moderate positive correlation with CD4^+^ BAL lymphocytes (*r* = .666, *p* = .035; Figure 3D), suggesting that the increased accumulation of Tregs and CD4^+^ T cells may act an important role in the increased secretion of TGF‐β1.

### 3.4 Comparison of the expression of chemokines on CD4^+^ T cells between the untreated and treated groups

**Figure 4:**
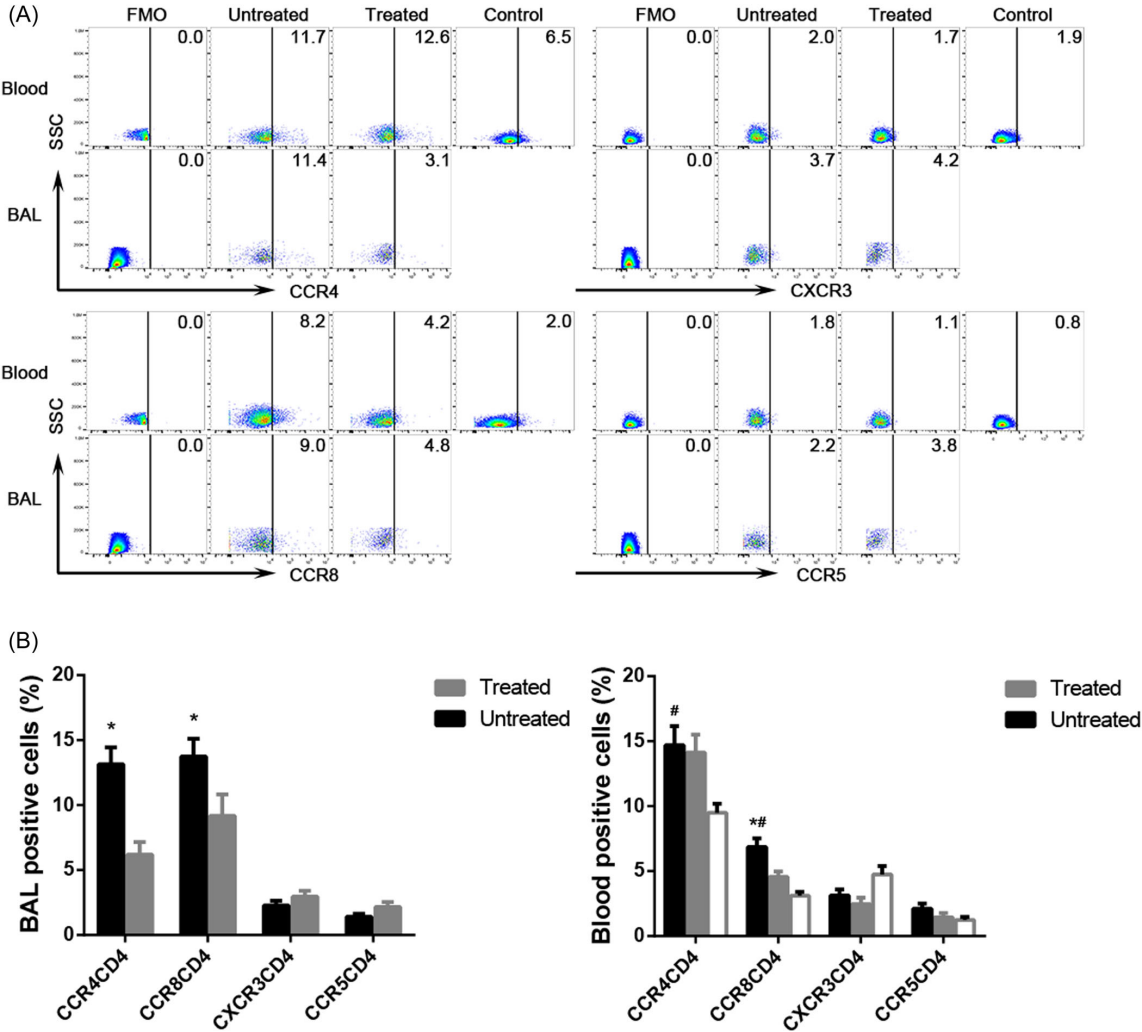
The expression of different chemokines on the CD4 surface in BAL fluid and peripheral blood of the untreated groups, treated groups, and control subjects. (A) Representative flow cytometric dot plots of CCR4, CCR8, CCR5, and CXCR3 on CD4^+^ T cells in BAL fluid, blood, and control. (B) Comparisons of percentages of CCR4, CCR8, CCR5 and CXCR3 on CD4^+^ T cells in BAL fluid and blood of the untreated groups, treated groups, and control subjects (*n* = 10). Data are expressed as the mean ± SEM. Within‐group comparisons before and after treatment were made using paired *t* tests or Wilcoxon's signed‐rank tests, and between‐group comparisons were analyzed using one‐way ANOVA followed by Newman–Keuls tests. *p < .05 compared with the treated groups, and # *p* < .05 com*p*ared with the control groups. BAL, bronchoalveolar lavage.

The untreated patients had significantly higher expression of CCR4 and CCR8 on BAL fluid and peripheral CD4^+^ T cells than the treated patients and the control subjects. A reverse trend was detected in BAL fluid and peripheral blood CD4CCR5 and CD4CXCR3 lymphocytes but without any statistical significance (for CD4CCR4, CD4CCR8, CD4CXCR3, and CD4CCR5 in BAL fluid, *p* = .004, *p* = .038, *p* = .235, and *p* = .129 vs. the treated patients; for CD4CCR4, CD4CCR8, CD4CXCR3, and CD4CCR5 in peripheral blood, *p* = .762, *p* = .001, *p* = .422, and *p* = .237 vs. the treated patients, *p* = .005, *p* = .000, *p* = .056, and *p* = .075 vs. the control subjects; Figure 4A,B). The above results suggest that the comprehensive treatment of TS tends to affect the expression of these receptors.

### 3.5 Comparison of the expression of chemokines on CD4^+^ T cells between BAL fluid and peripheral blood

**Figure 5:**
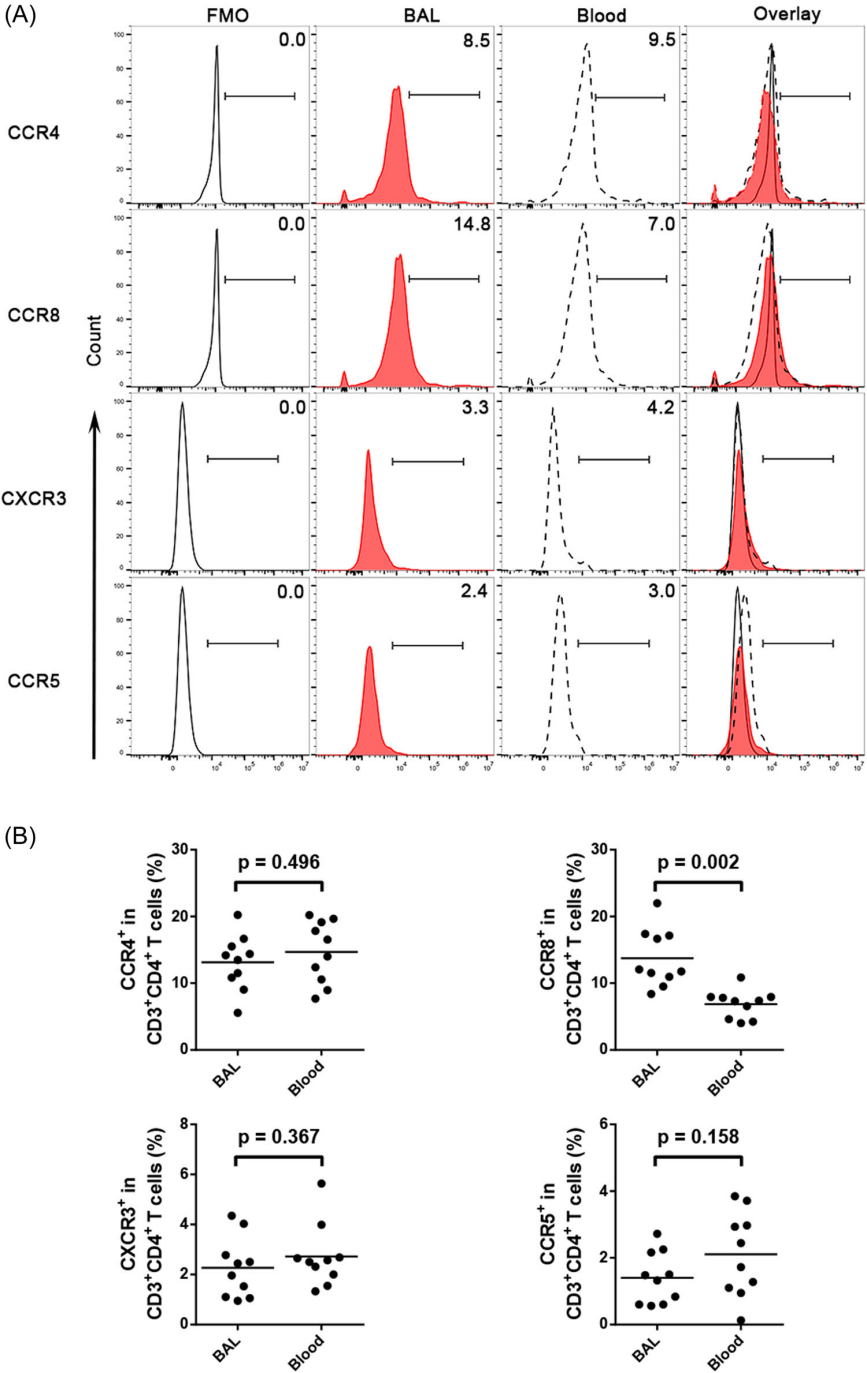
The percentages of CCR4, CCR8, CXCR3, and CCR5 on CD4^+^ T cells in BAL fluid and homologous peripheral blood. (A) Representative flow cytometric dot plots showing the expression of CCR4, CCR8, CXCR3, and CCR5 on CD4^+^ T cells in BAL fluid and blood. (B) Comparisons of percentages of CCR4, CCR8, CXCR3, and CCR5 on CD4^+^ T cells in BAL fluid and blood (*n* = 10). Horizontal bars indicate means. Comparisons were made using paired *t* tests or Wilcoxon signed‐rank tests.

The expression of CCR8 was significantly higher in BAL fluid than that in peripheral blood CD3^+^CD4^+^ T lymphocytes in the untreated patients, while CCR4, CXCR3, and CCR5 on CD4^+^ T cells showed an opposite trend without statistically significant differences (CCR8CD4 13.7 ± 1.4% vs. 6.9 ± 0.7%, *p* = .002, CCR4CD4 13.1 ± 1.3% vs. 14.7 ± 1.5%, *p* = .496, CXCR3CD4 2.3 ± 0.4% vs. 2.7 ± 0.4%, *p* = .367, CCR5CD4 1.4 ± 0.2% vs. 2.1 ± 0.4%, *p* = .158; Figure 5A,B).

### 3.6 Chemokine evaluation

**Figure 6:**
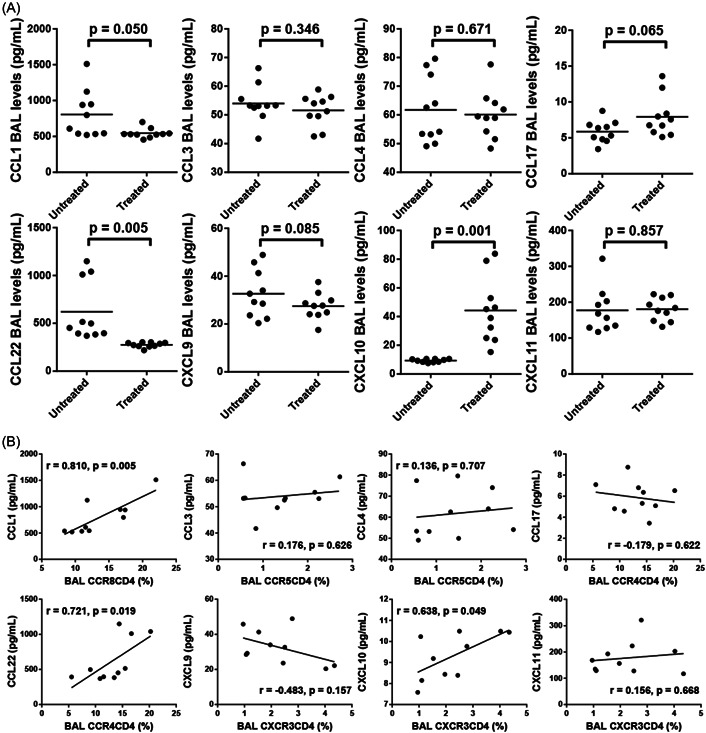
The expression levels of chemokines in BAL fluid and correlation analysis with chemokine receptors. (A) The statistical graph shows the concentration contrast of chemokines CCL1, CCL3, CCL4, CCL17, CCL22, CXCL9, CXCL10, and CXCL11 between the untreated and treated groups (*n* = 10). (B) Correlation analysis between chemokines and paired chemokine receptors. Horizontal bars indicate means. Comparisons were made using paired *t* tests or Wilcoxon signed‐rank tests, and correlations were determined by Spearman's rank correlation coefficients. BAL, bronchoalveolar lavage.

Taking into account all the patients without the comprehensive treatment, the levels of CCL1 and CCL22 in BAL fluid (CCR8 and CCR4 ligands, respectively) of the untreated patients were higher than those of the treated groups (*p* = .050 and *p* = .005, respectively; Figure 6A), while the concentration of CXCL10, one of the CXCR3 ligands, decreased significantly in the untreated groups (*p* = .001; Figure 6A). It was also observed that there were high expression levels of CXCL11 (177.4 ± 19.6 pg/mL vs. 180.4 ± 10.2 pg/mL, *p* = .857), medium expression levels of CXCL9 (32.7 ± 3.1 pg/mL vs. 27.5 ± 1.7 pg/mL, *p* = .085), CCL3 (54.0 ± 2.1 pg/mL vs. 51.6 ± 1.7 pg/mL, *p* = .346), and CCL4 (61.7 ± 3.7 pg/mL vs. 60.1 ± 2.6 pg/mL, *p* = .671), and low expression levels of CCL17 (5.9 ± 0.5 pg/mL vs. 7.9 ± 0.9 pg/mL, *p* = .065; Figure 6A) in BAL fluid of the untreated and treated groups.

As shown in Figure 6B, CCL1 BAL levels of the untreated groups were correlated with the expression of CCR8 on CD4 BAL lymphocytes (*r* = .810, *p* = .005), and CCL22 was positively associated with CCR4 expression on CD4 BAL cells (*r* = .721, *p* = .019). Additionally, there was a positive correlation between CXCL10 BAL levels and CXCR3 expression on CD4 BAL cells (*r* = .638, *p* = .047). No association was found between BAL levels of CCL17, CCL3, CCL4, CXCL9 or CXCL11 and CCR4, CCR5 or CXCR3 expression on CD4 cells.

**Figure 7:**
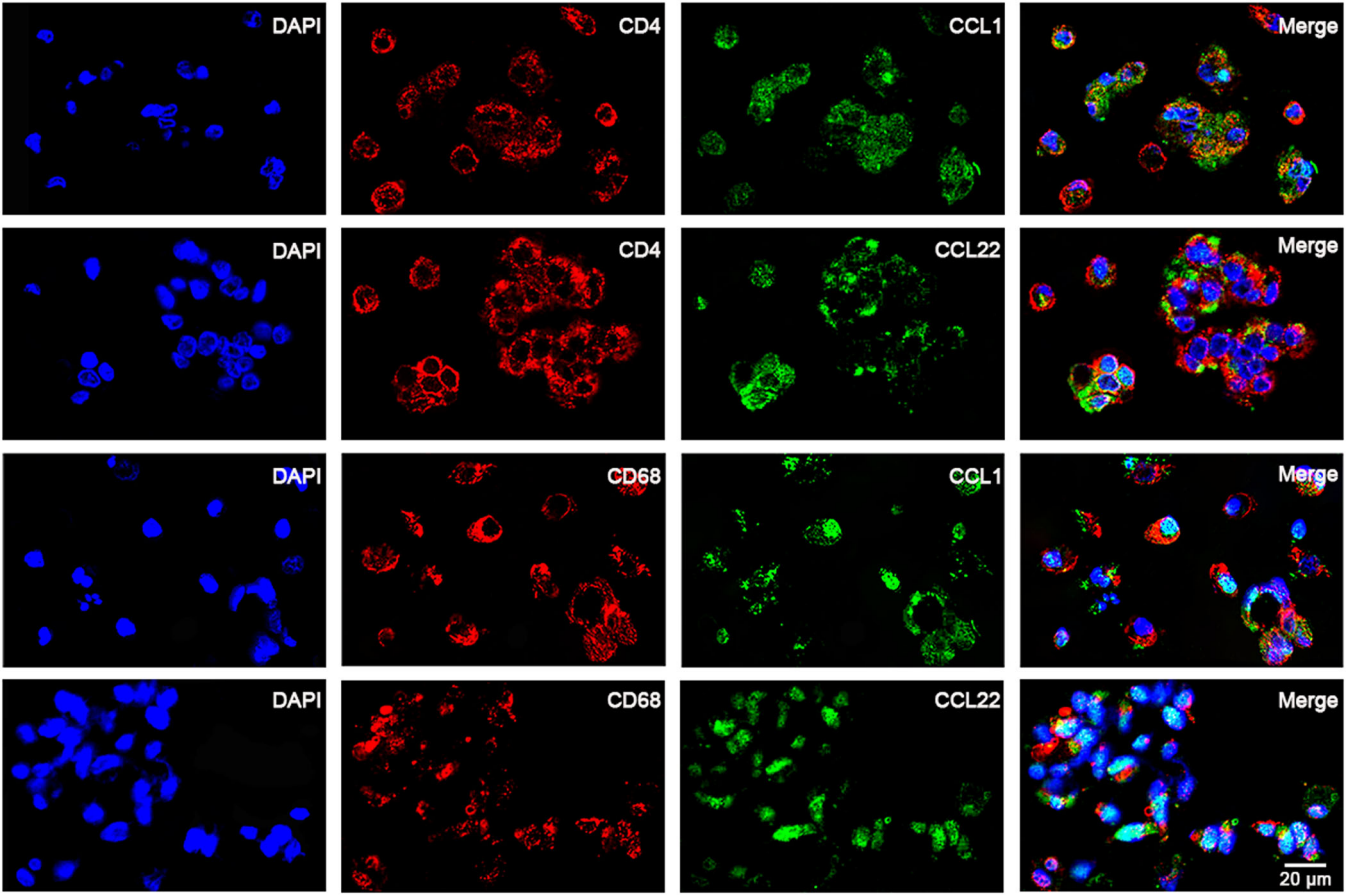
CCL1 and CCL22 were expressed in CD4^+^ T cells and CD68^+^ macrophages in TS BAL fluid. Macrophages and CD4^+^ T cells were tagged with anti‐CD68 and anti‐CD4 murine monoclonal antibodies, followed by rhodamine‐labeled goat antimouse serum staining (red). The expression levels of CCL1 and CCL22 were labeled with rabbit antihuman polyclonal antibodies and then labeled with FITC in goat antirabbit IgG (green). Two‐color immunofluorescence staining showed that some CD68^+^ macrophages and some CD4^+^ T cells expressed CCL1 and CCL22. Scale bar = 20 μm.

The previous ELISA analysis showed that CCL1 and CCL22 were highly expressed in BAL fluid and were highly positively correlated with their chemokine receptors. Thus, to visualize the distribution of chemokines in BAL fluid, double immunofluorescence staining was performed on cells isolated from BAL fluid, and it was discovered that CCL1 and CCL22 were expressed in CD4^+^ T cells and CD68^+^ macrophages (Figure 7). These data imply that CD68^+^ macrophages and CD4^+^ T cells may be the source of CCL1 and CCL22 in BAL fluid.

### 3.7 Chemokines CCL1 and CCL22 may be involved in the active recruitment of CD4^+^ T cells in TS

**Figure 8:**
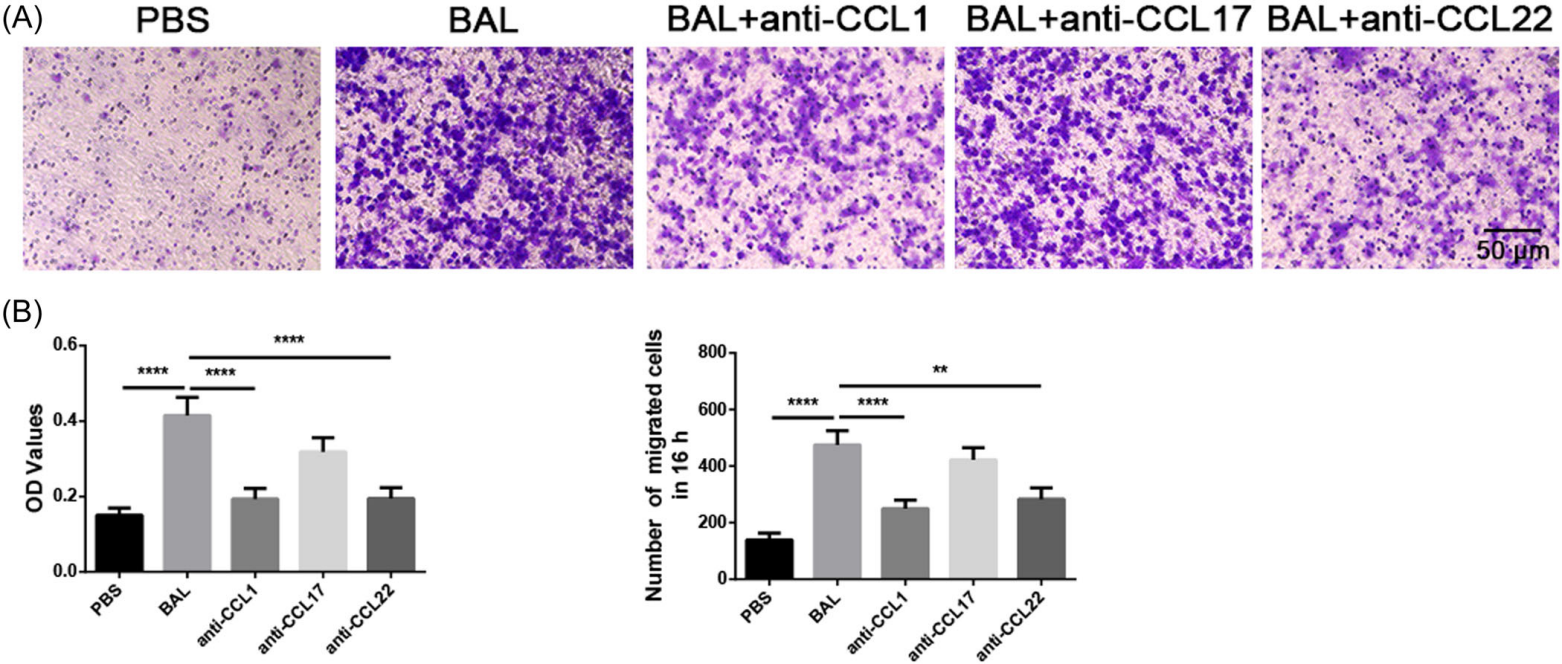
CCL1 and CCL22 in BAL of TS showed chemotactic activity against CD4^+^ T cells. The Transwell method was adopted for migration assay. Purified CD4^+^ T cells (1 × 10^5^) were plated into the upper chamber of a 6.5 mm Transwell (5 μm pore size). Three neutralizing antibodies (anti‐CCL1 0.1 μg/mL; anti‐CCL17 1 μg/mL; and anti‐CCL22 1 μg/mL) were added to the lower chamber. PBS was used as a negative control, and pure BAL was used as a positive control. After 16 h, CD4^+^ T cells on the upper side were removed, and cells in the lower chamber were fixed with methanol and stained with 0.1% crystal violet. (A) Representative microscopy pictures from six independent experiments, scale bar = 50 μm. (B) Chemotactic assay statistics analyzed by direct counting (number of migrated cells in 16 h) and indirect counting (optical density values). Data are expressed as the mean ± SEM. Between‐group comparisons were analyzed using one‐way ANOVA followed by Newman–Keuls tests. *p < .05, **p < .01, ***p < .001, and ****p < .000.

The aforementioned data indicated that CD4^+^ T cells in BAL fluid expressed high levels of CCR4 and CCR8, as well as elevated amounts of CCL22 and CCL1. Considering that the interactions between chemokines and their related chemokine receptors affect lymphocyte infiltration significantly,^41^ the CD4^+^ T cells that contribute to the local increase in the mechanism of TS may be the process of their active recruitment to CD4^+^ T cells. In vitro chemotaxis assays revealed that BAL fluid collected from the TS patients exerted chemotactic effects on peripheral CD4^+^ T cells through potential chemotactic activity. Anti‐CCL1 and anti‐CCL22 monoclonal neutralizing antibodies, but not anti‐CCL17, inhibited this chemotactic process significantly (Figure 8A,B), suggesting that the chemokines CCL1 and CCL22 may be key factors in the recruitment of CD4^+^ T cells from peripheral blood to infiltrate the diseased trachea in TS.

## 4 DISCUSSION

This study mainly focused on the comparison of indicators in the TS patients before and after treatment. Surprisingly, it found that the lung function, peripheral blood lymphocyte count, and blood gas analysis indexes of the TS patients effectively improved via transbronchoscopic intervention therapy and systemic anti‐infection treatment, and the inflammation indexes significantly reduced. Currently, transbronchoscopic intervention therapy has increasingly taken over as one of the primary treatments for treating benign airway stenosis,^5^ but the risk of restenosis is still high.^7^ Recent studies have found that tracheal transplantation has been considered a viable option for the reconstruction of long tracheal defects,^9^ while tissue engineering tracheal graft implantation may be the last chance for patients with severe cicatricial stenosis.^8^ At the same time, local and systemic anti‐infection has also been found to be effective for TS, but most of the data are related to animal models.^42^, ^43^, ^44^ The specific indicators of the clinical patients in this research fully demonstrated that comprehensive treatment, including bronchial intervention and systemic anti‐infection, could greatly improve various indicators of TS patients. However, it did not definitively distinguish which specific modality of treatment is more effective, nor did it refine the specific modality of bronchoscopy intervention. This will lay a foundation for our future detailed research.

In this work, despite the decrease of the total peripheral blood lymphocyte count in the untreated TS group, the results showed that compared with the treated patients and the healthy control subjects, there was a higher expression of CD4^+^ T cells and Tregs in tracheal tissue and BAL fluid of the untreated patients, and simultaneously their secreted TGF‐β1 expression was increased. These data were presented that supported the involvement of CD4^+^ T cells and Tregs in TS. Furthermore, TGF‐β1 expression in CD4^+^ T cells and Tregs in BAL fluid was strongly positively linked with those two cell types' expression in BAL fluid in TS.

The basic features of TS include excessive collagen production during tissue healing, as well as fibroblast differentiation and proliferation.^3^ The critical step in the repair process after tissue damage is the infiltration of immune cells.^45^ This study's experiment confirmed the hypothesis that there was a greater concentration of CD4^+^ T lymphocytes in the local area of TS. Studies have revealed that Tregs are a small subset of peripherally circulating CD4^+^ T cells that govern the immunological balance and are crucial for preserving health.^23^ In this study, however, a significant accumulation of Tregs were observed in the untreated TS patients. At the same time, the key cytokine TGF‐β1, which causes tissue fibrosis and is secreted by both CD4^+^ T cells and Tregs, particularly the latter, was abnormally elevated. This research proved a strong correlation between the secretion of TGF‐β1 and the number of these two immune cell subtypes, indicating that immune cells may be crucial in the onset and progression of TS through aberrant secretion of TGF‐β1 and other cytokines. Previous reports on TS fibrosis have mainly focused on the TGF‐β1 pathway.^46^, ^47^, ^48^ Therefore, this paper discussed the new possibility that the imbalance of immune cells and the secretion of cytokines may affect the occurrence and development of TS. At present, studies have constantly explored the potential of immune cells as targeted therapies. In animal models, T‐cellular immunotherapy can specifically target pathological mouse heart fiber vitamin cells to slow down the fibrosis process,^49^ so it is hoped that this indicator will become a targeted therapy for TS. However, this study did not delve into the mechanism of immune cell influence on TGF‐β1, which will be investigated in the next step.

Earlier studies have shown that Th1 cells are the primary source of INF‐γ, an inflammatory cytokine that can reduce fibroblast proliferation and collagen production,^29^ and thus Th1 cells inhibit fibrosis. Th2 cells that secrete IL‐4 have been shown to induce fibroblast aggregation and promote fibroblast activation and proliferation.^30^ In this study, Th2‐targeted CCR4 and CCR8 chemokine receptors, as well as the chemokine ligands CCL22 and CCL1, were shown to be considerably enhanced in the TS BAL fluid of the untreated group. It has been shown that CCL22 chemotactic CCR4^+^ macrophages contribute to fibrosis localization by releasing large amounts of cytokines and thus promote fibrosis. In turn, macrophages can further affect the function of CD4^+^ T cells.^50^ Conversely, despite a proportional drop in the Th1‐targeted CCR5 and CXCR3 chemokine receptors in the untreated group, no significant difference was identified, likely due to limited specimen size. Additionally, this study discovered that the levels of CXCL10, a chemokine that draws CXCR3‐positive cells, were decreased in the untreated patients. According to recent studies, CXCR3 and CXCL10 are strongly associated with the angiogenesis process.^32^, ^33^, ^51^ According to Tager et al.^52^ receiving bleomycin caused lung fibrosis in CXCL10‐deficient mice to worsen. Moreover, recent studies have shown that CXCR3 exerts an angiostatic effect mediated by alternative splicing of the CXCR3 gene (CXCR3‐B).^53^ The balance between angiostatic and angiogenic processes may change if CXC chemokines and their receptors become dysregulated during inflammatory events. The untreated TS subjects in this study had low levels of CXCR3, and CXCL10 expression may be beneficial to the local angiogenic activity of TS. Therefore, starting from chemokine receptors, this study discovered a possible local Th1/Th2 subpopulation imbalance in TS and also found that Th2 cells, which are associated with fibrosis, were elevated, while Th1 cells, which inhibit fibrosis, were decreased.

Besides, this paper focused on the chemotactic mechanism of CD4^+^ T cells. At present, it is a poorly understood mechanism for CD4^+^ T accumulation in TS, and it is speculated that the mechanism of increased CD4^+^ T content in TS may be related to active recruitment in the TS microenvironment. Chemotactic receptors are essential for T‐cell migration, but how they affect CD4^+^ T‐cell migration to local TS remains unclear. Therefore, this study explored whether CCL1 and CCL22 are related to the process of CD4^+^ T aggregation in the local area of TS. The findings showed that the untreated groups had considerably higher levels of CCL1 and CCL22 in their BAL fluid than the treated groups, and immunofluorescence suggested that CD68^+^ macrophages and CD4^+^ T cells may be the cellular sources of these chemokines. Moreover, the receptors corresponding to CCL1 and CCL22, namely CCR8 and CCR4, were obviously expressed on the surface of both TS and homologous peripheral blood CD4^+^ T cells. These data suggest that CCL1 and CCL22 in TS may be associated with local accumulation of CD4^+^ T cells. Subsequent in vitro chemotactic tests further confirmed that BAL in TS could recruit peripheral CD4^+^ T lymphocytes. In the untreated group, anti‐CCL1 or anti‐CCL22 monoclonal neutralizing antibodies dramatically inhibited the chemotactic activity of BAL in TS. Thus, the chemotactic effects of CCL1 and CCL22 may mobilize peripheral CD4^+^ T cells to infiltrate the TS region.

To summarize, our findings showed that a great accumulation of immune cells, including CD4^+^ T cells and their important subtype Tregs, accumulated in the untreated TS patients, and it was suspected that these cells might be involved in the fibrosis progression through the release of TGF‐β1. In this research, it was found that Th1/Th2 cells were locally imbalanced in TS, of which Th2 cells may play a role by actively recruiting TS through CCR4/CCL22 and CCR8/CCL1. However, after comprehensive clinical treatment, the phenotype of these immune cells changed. This study can serve as a breakthrough point for the future study of immune cells in TS fibrosis and lay a foundation for the exploration of TS immunotherapy.

## AUTHOR CONTRIBUTIONS

**Tingmei Feng**: Data curation; formal analysis; methodology; software; validation; writing—original draft. **Yan Chen**: Validation. **Jinmei Wei**: Methodology; validation. **Sen Tan**: Resources. **Liu Guangnan**: Writing—review & editing.

## CONFLICT OF INTEREST STATEMENT

The authors declare no conflict of interest.

## ETHICS STATEMENT

This study was approved by the ethics committee of the Second Affiliated Hospital of Guangxi Medical University [Approval number: 2022(KY‐0761)].
